# Disseminated Metacestode *Versteria* Species Infection in Woman, Pennsylvania, USA^1^

**DOI:** 10.3201/eid2507.190223

**Published:** 2019-07

**Authors:** Bethany Lehman, Sixto M. Leal, Gary W. Procop, Elise O’Connell, Jahangheer Shaik, Theodore E. Nash, Thomas B. Nutman, Stephen Jones, Stephanie Braunthal, Shetal N. Shah, Michael W. Cruise, Sanjay Mukhopadhyay, Jona Banzon

**Affiliations:** Cleveland Clinic Foundation, Cleveland, Ohio, USA (B. Lehman, G.W. Procop, S. Jones, S. Braunthal, S.N. Shah, M.W. Cruise, S. Mukhopadhyay, J. Banzon);; University of Alabama Medical Center, Tuscaloosa, Alabama, USA (S.M. Leal, Jr.);; National Institutes of Health, Bethesda, Maryland, USA (E. O’Connell, J. Shaik, T.E. Nash, T.B. Nutman)

**Keywords:** cestoda, parasite, agammaglobulinemia, Versteria, cestode, Taenia, tapeworm, parasites, Pennsylvania, metacestode, United States

## Abstract

A patient in Pennsylvania, USA, with common variable immunodeficiency sought care for fever, cough, and abdominal pain. Imaging revealed lesions involving multiple organs. Liver resection demonstrated necrotizing granulomas, recognizable tegument, and calcareous corpuscles indicative of an invasive cestode infection. Sequencing revealed 98% identity to a *Versteria* species of cestode found in mink.

In July 2017, a 68-year-old woman in Pennsylvania, USA, sought care for fever, fatigue, cough, and abdominal pain. Her medical history was significant for common variable immunodeficiency and splenic B cell lymphoma that had been treated with R-CHOP (rituximab, cyclophosphamide, hydroxydaunorubicin, vincristine, and prednisone); treatment was completed in December 2016. 

Imaging showed extensive nodular disease of the lungs and liver and a hepatic abscess. Examination of a fine-needle aspirate of the hepatic lesion detected hepatocytes with focal atypia on a background of marked acute inflammation and necrosis, suggestive of an active infectious process. Subsequent percutaneous needle biopsy samples of the liver, bronchoalveolar lavage and transbronchial biopsy samples, and surgical biopsy samples of the left lower lobe showed necrotizing granulomas and reactive/reparative tissue changes. All histochemically stained slides (Gomori-methenamine silver, Gram, periodic acid Schiff, Warthin-Starry, Ziehl-Neelsen, Fite) yielded negative results for microorganisms. Results of broad-range PCR for bacteria (16S rDNA), fungi (28S rDNA), and mycobacteria (16S rDNA, *rpo*B, and *hsp*65) were also negative.

Four months later, after the patient had been receiving broad-spectrum antibacterial and antifungal medications, she sought a second opinion at the Cleveland Clinic (Cleveland, OH, USA), where repeat cross-sectional imaging showed progressive nodular disease within the lungs, liver, and kidneys and cyst-like lesions in the eyes and brain (Appendix Figure 1). Gross examination of a liver sample from a right partial hepatectomy performed for diagnosis revealed multifocal tan-white nodules and necrotic or cystic spaces. Microscopic analysis identified extensive necrotizing granulomatous inflammation and multifocal cystic spaces, which enclosed material characteristic of the tegument of a cestode. In a separate location within otherwise nondescript necrotic tissue was a focal collection of round basophilic concretions with concentric layers of deposited material characteristic of calcareous corpuscles, pathognomonic for a cestode infection (Appendix Figure 2). Additional histochemical studies for microorganisms detected no microorganisms.

These findings were consistent with a disseminated proliferating invasive cestode infection; the metacestode most closely resembled the cysticercus larva that lacks a scolex (i.e., the racemose form of cysticercosis). The presence of racemose-like disseminated involvement of multiple visceral organs was concerning because this feature is not common in patients with cysticercosis. Results of an enzyme-linked immunotransfer blot for *Taenia solium* cestodes were negative. Cysticercus-specific IgG was not elevated, and antibodies against echinococci were not found, although these tests are unreliable in a patient who has common variable immunodeficiency and is receiving intravenous immunoglobulin. Therefore, we considered the possibility of another cestode species.

The patient received praziquantel and albendazole for 1 month. Initially, dexamethasone (10 mg) was concurrently administered for the neurologic and ocular involvement. Treatment resolved the abdominal pain, fatigue, and fever. Follow-up imaging showed vast improvement in the brain, lung, kidney, and liver lesions. Imaging findings continued to improve after corticosteroids were tapered off after 3 months, and symptoms continued to improve 6 months after treatment. However, serial eye examinations revealed a new cystic lesion in the eye. The cyst was extracted; histopathologic examination did not detect a scolex but did detect an identical tegument, again appearing as an aberrant form (Appendix Figure 2). As of April 2019, the patient was continuing to receive albendazole and praziquantel and monthly intravenous immunoglobulin.

Because of the unusual histopathologic findings and clinical course, we performed molecular analysis. We extracted DNA from formalin-fixed, paraffin-embedded liver tissue and then performed partial mitochondrial cytochrome (*cox1*) gene amplification. (*1*). PCR products were inserted into pCR 2.1 TOPO (https://www.thermofisher.com), cloned, and sequenced (at Macrogen USA, Rockville, MD, USA; https://www.macrogenusa.com). Our search for a 128-bp consensus sequence by using BLAST (https://blast.ncbi.nlm.nih.gov/Blast.cgi) found a 98% match to the *Versteria* species *cox1* gene (GenBank accession no. KT223034). After disease recurrence and soon after extraction of the ocular cyst, we subsequently subjected DNA from the preserved ocular cyst to Nanopore sequencing (Oxford Nanopore Technologies, https://nanoporetech.com) and assembled the complete mitochondrial genome, which we deposited at GenBank (accession no. MK681866) (Figure).

**Figure:**
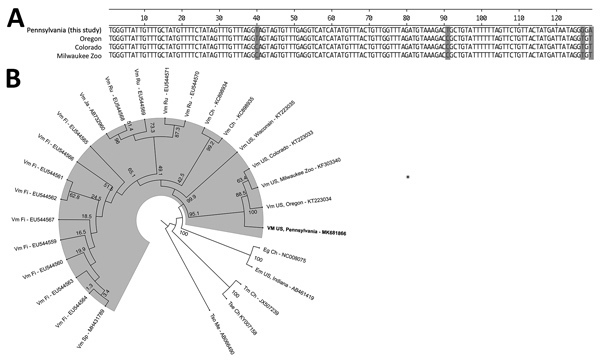
Test results for woman with disseminated *Versteria* sp. cestode infection, Pennsylvania, USA. A) Sequence of the 129-bp fragment of cytochrome c oxidase subunit 1 (*cox1*) gene from patient compared with 3 closely related *Versteria* sp. isolates from the United States. Shading indicates differing nucleotides. B) Phylogenetic tree based on the *cox1* gene of all reported Vm *cox1* sequences with country of origin and other clinically relevant cestodes; GenBank accession numbers are provided. Boldface indicates the Vm sequence reported in this study; shading represents the outbranching of Vm. Bootstrap values are shown. Ch, China; Eg, *Echinococcus granulosus*; Em, *Echinococcus multilocularis*; Fi, Finland; Ja, Japan; Me, Mexico; Ru, Russia; Sp, Spain; Tm, *Taenia multiceps*; Tse, *T. serialis*; Tso, *T. solium*; US, United States; Vm, *Versteria* sp. mitochondrial.

The definitive hosts of the new *Versteria* (*Taenia mustelae*) cestodes are usually mustelids (*2*), a family of carnivorous mammals including weasels, ermine, mink, and others, which are found throughout the northern United States (*3*). This patient reported exposure to fishers in her residence in western Pennsylvania, where a resurgence in the population of these members of the family Mustelidae has been observed. Her husband was screened for signs of a parasitic infection and results were negative. The only other reported human infection with *Versteria* sp. involved a kidney transplant patient, who also had lung and liver lesions. Histopathologic examination of that patient’s liver lesions revealed focal necrotizing granulomas with hooklets and a protoscolex (*4*).

The diagnosis of a cestode infection is usually suggested by the presence of specific cestode structures (e.g., a protoscolex, tegument, or calcareous corpuscles). However, unlike the previous report of human infection, histopathologic examination of the liver lesion and ocular cyst from this patient did not detect hooklets or protoscoleces, mimicking the histopathologic appearance of racemose disease sometimes seen in patients with subarachnoid neurocysticercosis. Because histopathologic examination is insufficient for species-level identification (specific cestode structures), molecular testing is necessary for definitive diagnosis of *Versteria* sp. cestode infection. 

Appendix Images and histopathology slides from woman with *Versteria* sp. infection, Pennsylvania, USA.
