# Supplementary material for: Disseminated Metacestode Versteria Species Infection in Woman, Pennsylvania, USA
Source: Emerg Infect Dis. 2019 Jul;25(7):1429–31. doi: 10.3201/eid2507.190223 (PMC6590738; doi:10.3201/eid2507.190223)
Supplement: Appendix — Images and histopathology slides from woman with Versteria sp. infection, Pennsylvania, USA. [file 19-0223-Techapp-s1.pdf]

Article DOI: https://doi.org/10.3201/eid2507.190223

Disseminated *Versteria* Sp. Metacestode Infection in Woman, Pennsylvania, USA

Appendix

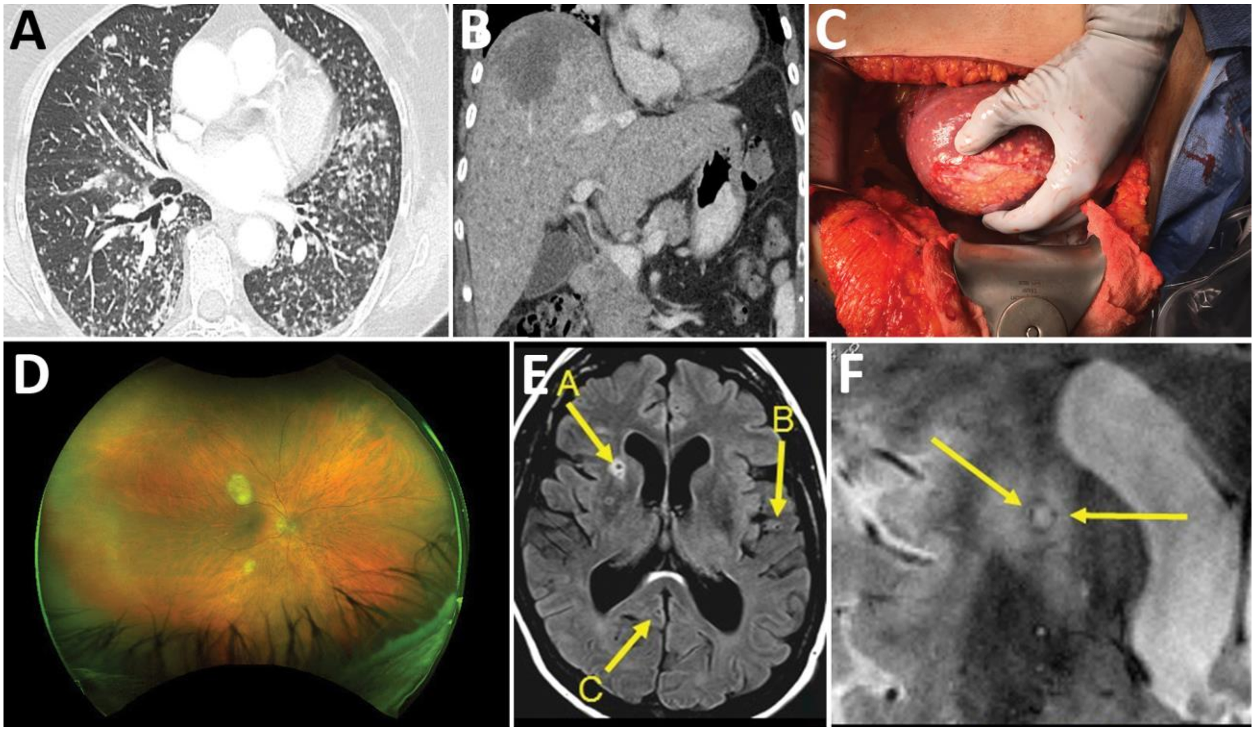

Appendix Figure 1. Images of patient with *Versteria* sp. infection. A) Contrast-enhanced CT scan of the chest showing numerous randomly distributed sub-centimeter nodules throughout both lungs with predominant involvement in mid to lower lungs. B) Coronal reformatted image from contrast enhanced CT scan of the abdomen demonstrating a low attenuation right hepatic dome lesion measuring 6.0 × 4.7 cm, numerous smaller low attenuation lesions in the liver. C) Visualization of the liver at surgery revealing multifocal tan-white nodules and cystic spaces. D) Wide field fundus photo which reveals 2 oval subretinal lesions in the right eye. E) Axial FLAIR image from 7T MRI, showing three example cystic lesions: (A) right anterior limb of the internal capsule; (B) left temporal operculum; and (C) right posterior cingulate gyrus. Lesion A demonstrates mild peri-lesional edema. Numerous other lesions were found throughout the brain (not shown). F) Magnified view of lesion A in 1e, using a high resolution T2* GRE sequence, showing voids (dark signal) at lesions periphery representing calcification or iron in the wall of the lesion.

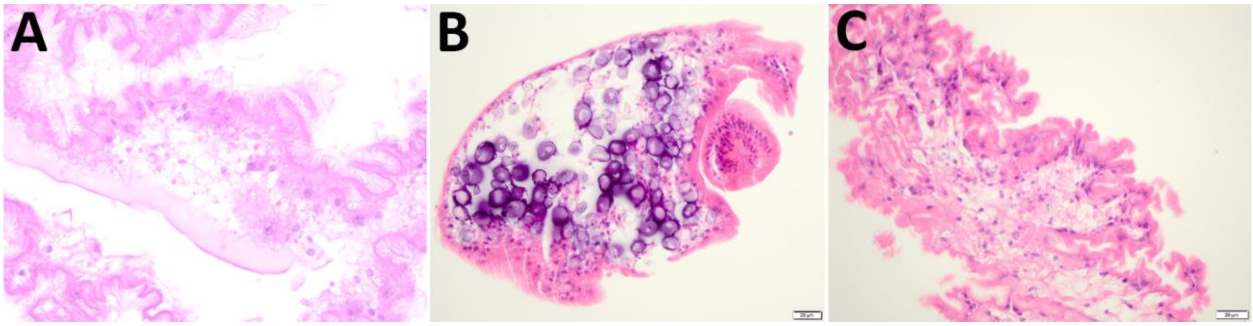

Appendix Figure 2. Histopathology images of tissue from patient with *Versteria* sp. infection. A) Degenerating three-layered membrane characterized by a undulating, eosinophilic outer cell layer with microvilli underlying degenerating cells with pyknotic nuclei (i.e., pyknotic cell layer), and degenerating loose connective tissue from the open liver biopsy. B) Numerous calcareous corpuscles (ie purple structures) and protoscolex obtained from the ocular cyst extraction. C) Ocular cyst demonstrating the three-layered membrane again with outer layer with microvilli, pyknotic cell layer and loose connective tissue.
